# Incidence of Malocclusion among Young Patients with Gingival Recessions—A Cross-Sectional Observational Pilot Study

**DOI:** 10.3390/medicina57121316

**Published:** 2021-11-30

**Authors:** Darius Tomina, Smaranda Buduru, Cristian Mihail Dinu, Andreea Kui, Cătălina Dee, Raluca Cosgarea, Marius Negucioiu

**Affiliations:** 1 Department of Periodontology, University of Medicine and Pharmacy “Iuliu Hațieganu”, 400012 Cluj Napoca, Romania; dariustomina@gmail.com; 2 Department of Prosthetic Dentistry, University of Medicine and Pharmacy “Iuliu Hațieganu”, 400012 Cluj Napoca, Romania; ralucacosgarea@gmail.com (R.C.); negucioiu.marius@gmail.com (M.N.); 3 Department of Surgery and Maxillo-Facial Implantology, University of Medicine and Pharmacy “Iuliu Hațieganu”, 400012 Cluj Napoca, Romania; dinu_christian@yahoo.com; 4 Department of Maxillo-Facial Surgery, County Emergency Hospital, 400000 Cluj Napoca, Romania; deecatalina@yahoo.com; 5 Clinic of Periodontology, Operative and Preventive Dentistry, University of Bonn, 53111 Bonn, Germany; 6 Clinic of Periodontology and Peri-Implant Diseases, University of Marburg, 35033 Marburg, Germany

**Keywords:** lateral guidance, protrusion, gingival recession, malocclusion, occlusal trauma, working-side interferences

## Abstract

*Background and Objectives*: Dental occlusion and gingival recession have been studied over the past years especially because of the increasing incidence of occlusal interferences in young patients. The purpose of this pilot study is to investigate any association between occlusal dysfunctions and gingival recessions. Data on gingival phenotype and previous orthodontic treatment were also collected to assess any correlation with the presence of gingival recession. *Materials and Methods*: Forty systemically healthy subjects, without signs of periodontitis and with gingival recessions, were included in the study. The following parameters were determined: location and extent of the gingival recession, gingival phenotype and functional occlusion by means of observing and registering the occlusal contacts in maximum intercuspation position, protrusive and lateral guidance. *Results*: Premolars were mostly affected in cases of working-side interferences during lateral guidance (71.19% of the affected teeth during left and 75% during right mandibular movements). The chi-squared exact test applied for the analysis of contingency tables revealed statistically significant associations between excursive interferences during lateral guidance and anterior guidance and the presence of gingival recession on the involved group of teeth. *Conclusions*: The results suggest that most gingival recessions might be associated with working-side interferences, the highest number of gingival recessions being associated with active interferences during lateral guidance.

## 1. Introduction

Gingival recessions are defined as apical displacement of the gingival margin beyond the cementoenamel junction; they appear either as a response against local irritating factors (such as biofilm, calculus, oral piercing, etc.) or as incapacity of the gingival mucosa to adapt to excessive occlusal forces [1]. Often, gingival recessions cannot be associated with a single etiologic factor, which makes the clinical diagnostic and treatment even more difficult [2]. Several factors, such as age, oral hygiene, or gingival phenotype, may influence the incidence and progression of gingival recessions. While a thin gingival phenotype or insufficient height of the attached gingiva can make the periodontium vulnerable to various mechanical local injuries, evidence suggests that occlusal trauma may act as a trigger factor in cases with a deficient anatomical background (i.e., thin buccal bone, decreased vestibular depth, aberrant frenulum and muscle position, inconsistent gingival margin and tooth dimension) [3,4,5].

Occlusal relationships of maxillary and mandibular arches are of paramount importance in dentistry. Whether it is a fully dentate patient or a complex situation of complete mouth rehabilitation, the clinical analysis of dental occlusal requires time and precision, for both static and dynamic occlusion [6]. Several published studies regarding dental occlusion are inconsistent in the matter, probably due to the high number of definitions and their various meanings. The terms we used in this paper are the following: dental occlusion refers to each static contact between one or more lower teeth with one or more upper teeth [6]. Maximum intercuspation position (MIP) refers to the occlusal interrelationship among upper and lower teeth, where there is maximum activity of the muscles and is determined by the shape and position of teeth, periodontal proprioceptors, muscle memory and occlusal contacts [7].

Anterior guidance is the dynamic relationship among the lower anterior teeth and the upper anterior teeth through protrusive excursions. Lateral guidance refers to the dynamic relationship among lower teeth and upper teeth in lateral excursions; canine guidance or group function are considered physiologic situations in case of lateral excursions [8]. During lateral excursions, working side refers to the side toward which the movement is performed, while non-working side/balancing side refers to the side opposite to the movement. Excursive interferences refer to protrusive interferences, as well as interferences of the working side; as for the interferences of the non-working side in case of lateral guidance, they are referred to as balancing interferences [6].

Occlusal trauma refers to injuries determining tissue changes within the attachment apparatus, including periodontal ligament, supporting alveolar bone and cementum, as a result of occlusal forces [9,10]. Occlusal trauma may occur on teeth with a normal periodontium or with reduced periodontal support [9]. Primary occlusal trauma (i.e., active or passive interferences, excessive occlusal load) can lead to tooth mobility, tooth crack, temporomandibular disorders (TMDs) and abfraction of teeth’s hard tissues [11].

Dental occlusion and gingival recessions have been studied over the past years, especially with an increasing incidence of occlusal interferences in young patients. Moreover, patient’s expectations regarding pink–white esthetics have increased [10,11]. Abnormal occlusal forces can determine a major impact on tooth and periodontium to varying degrees or they do not affect these structures at all due to adaptive capacity of the stomatognathic system. An important role in the adaptive capacity is played by gingival thickness. A thick gingival phenotype can postpone or delay the processes, while a thin phenotype associated with the absence of attached gingiva is considered a risk factor for the development of gingival recessions [12,13,14].

Several classifications of gingival recessions are reported in the literature. Cairo’s classification (2011), based on the assessment of the clinical attachment level at both the buccal and interproximal sites, proposes three situations: (1) recession type 1 (RT1)—no loss of interproximal attachment; (2) recession type 2 (RT2)—gingival recession associated with loss of interproximal attachment, in a lower or equal amount compared to the buccal attachment; (3) recession type 3 (RT3)—gingival recession associated with loss of interproximal attachment, in a higher amount than the buccal attachment [15]

The hypothesis of the study was that gingival recession that occurred on a certain group of teeth, irrespective of the gingival phenotype, can be correlated with occlusal interferences. In this context, the following questions are addressed: (1) Is there a relationship between occlusal interferences and gingival recessions? (2) Does the gingival phenotype influence the development of gingival recession in patients with occlusal dysfunction? (3) Which group of teeth would be more prone to gingival recession in case of occlusal interferences?

The present study aims to evaluate the incidence of occlusal interferences in young patients with gingival recession and no other signs of gingival inflammation or periodontitis and determine a potential relationship between occlusal interferences and gingival recession.

## 2. Materials and Methods

### 2.1. Design and Settings

Eighty-two healthy subjects were selected for participating in our research study; all of them were informed about the examination protocols and all signed the informed written consent. The inclusion criteria were as follows: (1) age, between 20 and 35 years old; (2) Angle’s class 1 occlusal relationships; (3) full dentition except for third molars; (4) no ongoing orthodontic therapy; (5) at least one gingival recession ≥1 mm with no interproximal attachment loss (RT1); (6) no other clinical signs of gingival inflammation or supragingival calculus; (7) intact cementoenamel junction (CEJ) for the examined teeth. The exclusion criteria were as follows: (1) gingival inflammation; (2) smoking habit; (3) ongoing orthodontic treatment; (4) incorrect direct or direct restorations in violation of the biological width; (5) non-carious cervical lesions; (6) diagnostic of temporomandibular disorders; (7) Angle’s class II or III; (8) recession types 2 or 3.

### 2.2. Materials

The examination procedures were performed by two experienced specialists and consisted of a general anamnestic evaluation, followed by a periodontal and occlusal examination, using an examination kit, a periodontal probe PCP UNC-15 (HuFriedy^®^, Chicago, IL, USA), 200 µm and 40 µm red/blue articulating paper (Bausch^®^, Nashua, NH, USA), shimstock occlusal registration strips 12 µm thick (Arti-Fol metallic articulating film; Dr. Jeau Baugh KG, Köln, Germany) and Miller forceps.

Periodontal evaluation consisted in localizing, classifying and quantifying the gingival recession—measuring the distance between the cementoenamel junction (CEJ) and the most apical part of the free gingival margin of the teeth and recording the value in mm in the patient’s chart. The Cairo classification was used for rating gingival recession. The gingival phenotype was determined by the mucosal transparency probe technique, using the same periodontal probe. The probe was gently inserted into the gingival sulcus of the examined teeth and gingival transparency was evaluated. The gingival phenotype was classified as thin if the examiner was able to see the probe through the gingival margin and thick if the examiner was unable to see the probe trough the tissues.

Occlusal evaluation consisted in recording the contact points between antagonistic arches in maximum intercuspation MIP and eccentric movements (anterior guidance and lateral guidance). To determine the MIP, 200 µm blue articulating paper was used and the distribution and intensity of the marks was analyzed. The articulating paper was held intraorally (using Miller forceps) while the subject tapped their teeth together firmly through the articulating paper 5 times in succession, through a single marking strip. Each subject was instructed to attempt to generate their perceived maximum occlusal force while tapping through the articulation strips. The presence or absence of contact points was additionally checked with 12 µm shimstock articulating paper. The Angle class of the patient in MIP was also determined.

During lateral guidance, canine and group guidance were considered functional; excursive interferences occurred when either 1 or 2 posterior teeth on the same side were involved (in canine guidance) or when 1 posterior tooth was more strongly marked (in group function). Balancing-side interferences were considered when posterior teeth on the opposite side did not disocclude. The interferences were assessed with shimstock and marked with 40 µm red articulating paper.

During anterior guidance, at least one pair of incisors (maxillary and mandibular), on each side of the midline associated with the immediate disocclusion of posterior teeth, was considered functional. Excursive interferences were considered when only one pair of teeth guided the movement, or all protrusive contacts were distributed only one side of the midline, as well as when the marks’ intensity differed between the guiding teeth. Posterior interferences were considered when disocclusion did not occur immediately between posterior teeth, right after the mandible left the centric relation.

The markings resulted during MIP or excursive movements were photographed off a mirror using a digital SLR camera (Nikon D3100, Nikon Inc., Melville, NY, USA), using an aperture of F32 and shutter speed of 2/5 s. For each patient, a photo portfolio was obtained, where all markings were easily discernable. Afterwards, two examinators evaluated each photograph in order to identify the more intense and large marks (considered pathological) compared to the rest, as well as a marking on the inclines, meaning a contact point outside the dental fossa or marginal ridge, which would generate mandibular instability.

### 2.3. Statistical Analysis

The statistical analyses were performed using IBM^®^ SPSS^®^ Statistics 25.0 (IBM Inc., New York, NY, USA) and the Microsoft Excel application. The data are presented as mean ± standard deviation of the mean (SD). The qualitative analysis of the variables was performed using the chi-squared test, Fisher’s exact test and the Z test. The variations in the results were considered statistically significant if *p* < 0.05.

## 3. Results

Out of the 82 subjects examined, 40 (16 males and 24 females; age of 24.62 ± 1.03 years) were further included in this pilot study, based on the inclusion and exclusion criteria (Table 1). In total, 27 subjects (67.5%) showed excursive interferences and 6 subjects (15%) showed posterior interferences during protrusive mandibular movement. During right lateral guidance, 26 subjects (65%) presented excursive interferences and 4 (10%) showed balancing interferences. During left lateral guidance 22 subjects (55%) presented excursive interferences and 3 (7.5%) presented balancing interferences (Table 2).

In total, 16 out of the 27 subjects presenting excursive interferences in anterior guidance displayed gingival recession localized on the guidance teeth. In addition, three subjects out of the six presenting posterior interferences displayed gingival recessions.

A total of 20 out of the 26 subjects presenting working-side interferences (in right lateral guidance) displayed gingival recession on the guidance teeth, while 17 out of the 22 subjects with working-side interferences (in left lateral guidance) displayed gingival recessions. Two out of the four subjects with balancing-side interferences also had gingival recessions (Table 3).

Among the 16 subjects with excursive interferences in protrusion, 9 of them presented a thin gingival phenotype, while 7 had a thick phenotype. For the subjects with excursive interferences in lateral guidance, 18 had a thick gingival phenotype compared with 19 with a thin phenotype. Two out of four subjects with balancing interferences in lateral guidance had a thin gingival phenotype (Table 3).

The chi-squared exact test applied for the analysis of contingency tables revealed statistically significant associations between excursive interferences during lateral guidance and anterior guidance and the presence of gingival recession on the involved group of teeth. No statistically significant associations were identified between gingival recessions and balancing interferences in lateral guidance or posterior interferences in anterior guidance (Table 4).

## 4. Discussion

The effects of occlusal trauma and excessive occlusal forces on the periodontium are still a controversial subject in periodontology. According to Glickman, trauma from occlusion is defined and diagnosed based on the histologic changes in the periodontium. In addition, Glickman clearly stated that, as long as the periodontium adapts to the occlusal relationships (even malocclusion) without loss of tooth-supporting tissues, a diagnostic of occlusal trauma cannot be confirmed [16,17]. However, insufficient stimulation of the periodontium might also injure the supporting periodontal tissues, by thinning the periodontal ligaments, causing fibers atrophy associated with osteoporosis of the alveolar bone and reduction in bone height [17].

The aim of our study is to evaluate the incidence of occlusal interferences in young patients with gingival recession, in the absence of any gingival inflammation or periodontitis and determine whether there might be a connection between occlusal interferences and gingival recession.

The statistical analyses revealed that a high percentage of gingival recessions (62.5%) were associated with excursive interferences, especially during right lateral guidance. For the anterior guidance, central upper and lower incisors were equally affected, as the lateral incisors tended to be less affected. During lateral guidance, the premolars involved in excursive interferences were mostly affected by gingival recessions (75% in case of right lateral guidance and 72% in case of left lateral guidance). In lateral guidance, the incidence of gingival recessions was low (12%) in case of balancing interferences and usually localized in the posterior area (Table 2).

Several studies revealed similar results. Krishna et al., in their study published in 2013, a selective sample of 50 subjects with gingival recessions and 10 subjects who had gingival clefts (mean age of 22) were examined in static and dynamic occlusion. The authors concluded that the large majority of subjects showed excursive interferences, while gingival recessions were more frequently related to the group function in lateral guidance, rather than canine guidance. For cases with canine protection in lateral guidance, gingival recessions were observed on the buccal surfaces, while, for group protection, gingival recessions were distributed on the lingual surfaces of the anterior teeth. The authors also emphasized how occlusal interferences, in the case of dynamic occlusion associated with an absence of mutually protected occlusion, may contribute to gingival lesions, such as gingival recessions and fissures [18].

In their study published in 2019, Kundapur et al. concluded that there was no association between the presence of gingival recession and occlusal interferences. While their group size was larger than that in our research study, with similar inclusion criteria, the parameters evaluated were limited to the frontal mandibular area. There is a risk a bias due to the small sample size taken in consideration for our study; however, the results based on our research work might indicate a strong association between gingival recession on the premolar area and eccentric lateral movements of the mandible [19].

We also want to emphasize the fact that our study does not provide information to support the hypothesis that patients with a thin phenotype are more exposed to gingival recession; on the contrary, the results revealed that patients with a thick phenotype were diagnosed with gingival recession and occlusal interferences in a similar percentage compared to the subjects with thin phenotype.

In our pilot study, the method used for occlusal analysis was the conventional method, consisting of the use of articulating paper. While several studies consider the method subjective, as it depends on the operator’s interpretation [20,21,22], shimstock foil combined with articulating paper markings has been widely used in the determination of occlusal tooth contacts that require adjustments [23]. As shimstock foil does not mark the selected teeth, the articulating paper markings guide the operator when selecting which contacts might be involved in interferences. To this matter, several textbooks on occlusion advocate that marked areas by the articulating paper are representative of the load contained within the mark [24,25,26]. In their study, Majithia et al. (2015) revealed no differences between digital analysis of occlusion (using TScan III) and the articulating paper markings in normal and treated maxillofacial trauma [27]. In another clinical study, Brizuela-Velasco et al. (2015) aimed to evaluate whether articulating paper thickness was related to the area of the occlusal contact registered. They concluded that the peripheral area of the occlusal registration with a higher chromatic intensity that was obtained using a thick articulating paper should not be eliminated during occlusal adjustments. In addition, the central area of the registration with a lower chromatic intensity is the real occlusal contact and correlated with the results obtained using a thinner articulating paper [28].

It is very difficult to isolate a single trigger point for the development of gingival recession and, to this matter, several articles reported that gingival recession was more likely to occur in patients who underwent orthodontic treatment [29,30,31]. However, our research work did not show any statistically significant difference between the subjects with or without orthodontic treatment prior to examination.

Our study might lay the foundation for future research focused on the correlation between occlusal interferences during lateral movements and gingival recessions in the premolar region, in young population without periodontal infectious disease.

We strongly believe there is a strong association between recession and working-side (excursive) interferences, especially for the lateral movements, aspect indicated by the preliminary data of our research. The lack of literature evidence may be due to the absence of uniformity regarding case definition, as well as proper diagnostics of the occlusal interferences and gingival recessions.

Nevertheless, the limitations of our pilot study arise also from the analog method used for occlusal examination. There are several clinical studies suggesting the importance of using digital devices (such as Tscan^®^ systems) for occlusal analysis, as those systems are reliable and less prone to subjective interpretation [20,21,22,23,24,25,26,32]. We are aware that the digital revolution has supplied the clinician with an upgradable product and periodic updates could remove some postprocessing faults and introduce new functionalities, resulting in a more exact final restoration in the future [33].

The findings of our pilot research study suggest that further larger clinical studies, investigating the association between occlusal trauma and gingival recession are required, with the use of digital systems in order to reduce or to eliminate the subjective variables.

## 5. Conclusions

Within the limitations of our study, we can conclude that gingival recessions were present in a high percentage in cases of excursive interferences, especially in lateral guidance. Passive interference caused recessions less frequently, although in those cases, they were evenly distributed throughout the teeth affected. During the active interferences in the lateral guidance movements, it was observed that the premolars are the most affected teeth. In passive interference of the protrusive movement, the mandibular premolars are affected to a greater extent than the maxillary premolars. Molars are the most exposed teeth to passive interferences in laterotrusive movements.

Despite the uncertainty about the true value of gingival thickness, the present results demonstrate that the gingival phenotype did not influence the occurrence of gingival recession associated with excursive/balancing-side interference in young patients.

We strongly believe that, in the coming years, the number of patients with gingival recession will raise along with the need for predictable and effective treatments. Therefore, a proper diagnostic of gingival recession should include the primary and secondary causes, as the treatment should focus on all factors that contributed to the trigger and progression of such pathology. While the results of our study indicate that there might be a connection among the factors investigated, further randomized clinical trials are needed in order to identify a strong association between occlusal trauma and gingival recession.

## Figures and Tables

**Table 1:** Data demographic distribution.

Demographic Categories	Frequency	Valid Percentage
Gender		
Female	16	40%
Male	24	60%
Age		
23–24	15	37.5%
25–26	25	62.5%
Previous orthodontic treatment		
Yes	11	27.5%
No	29	72.5%
Gingival phenotype		
Thin	19	47.5%
Thick	21	52.5%

**Table 2:** Occlusal interferences and gingival recession distribution on teeth type.

	PEI		PBI		LLWI		LLBI		RLWI		RLBI	
No. of teeth with recession/interference	57		13		59		5		72		10	
Max. central incisor	22	38.60%	-	-	3	5.08%	-	-	2	2.78%	-	-
Max. lateral incisor	4	7.02%	-	-	2	3.39%	-	-	2	2.78%	-	-
Mand. central incisor	21	36.84%	-	-	4	6.78%	-	-	1	1.39%	-	-
Mand. lateral incisor	10	17.54%	-	-	2	3.39%	-	-	3	4.17%	-	-
Max. premolars	-	-	4	30.77%	20	33.90%	-	-	27	37.50%	2	20%
Mand. premolars	-	-	5	38.46%	22	37.29%	-	-	27	37.50%	2	20%
Max. molars	-	-	2	15.38%	3	5.08%	2	40%	5	6.94%	3	30%
Mand. molars	-	-	2	15.38%	3	5.08%	3	60%	5	6.94%	3	30%

Protrusive excursive interference (PEI); protrusive balancing interference (PBI); right lateral working interference (RLWI); right lateral balancing interference (RLBI); left lateral working interference (LLWI); left lateral balancing interference (LLBI).

**Table 3:** Data distribution of interference type associated with gingival recession.

Occlusal Interference Type (Abbreviation)	% of Total	% (no.) of Recession + Guidance Teeth	Gingival Phenotype	
			Thin	Thick
Protrusive excursive interference (PEI)	67.5% (27)	59.25% (16)	56.25% (9)	43.75% (7)
Protrusive balancing interference (PBI)	15% (6)	50% (3)	66.66% (2)	33.33% (1)
Right lateral working interference (RLWI)	65% (26)	76.92% (20)	55% (11)	45% (9)
Right lateral balancing interference (RLBI)	10% (4)	50% (2)	50% (2)	50% (2)
Left lateral working interference (LLWI)	55% (22)	77.27% (17)	41.17% (7)	58.82% (10)
Left lateral balancing interference (LLBI)	7.5% (3)	66.66% (2)	50% (1)	50% (1)

**Table 4:** Contingency tables and *p*-value for chi-squared test.

	Rec +	Rec −			Rec +	Rec −			Rec +	Rec −	
PEI +	16	11	27	PBI +	3	3	6	RLWI +	20	6	26
PEI −	13	34	47	PBI −	34	7	41	RLWI −	14	20	34
	29	45	74		37	10	47		34	26	60
*p* = 0.012				*p* = 0.724				*p* = 0.008			
	Rec +	Rec −			Rec +	Rec −			Rec +	Rec −	
RLBI +	2	2	4	LLWI +	17	5	22	LLBI −	2	1	3
RLBI −	36	38	74	LLWI −	18	23	41	LLBI −	37	38	75
	38	40	78		35	28	63		39	39	78
*p* > 1				*p* = 0.001				*p* > 1			

Protrusive excursive interference (PEI); protrusive balancing interference (PBI); right lateral working interference (RLWI); right lateral balancing interference (RLBI); left lateral working interference (LLWI); left lateral balancing interference (LLBI).

## References

[1] Cortellini P., Bissada N.F. (2018). Mucogingival conditions in the natural dentition: Narrative review, case definitions, and diagnostic considerations. J. Periodontol..

[2] Addy M., Hunter M.L. (2003). Can tooth brushing damage your health? Effects on oral and dental tissues. Int. Dent. J..

[3] Harrel S.K., Nunn M.E. (2004). The effect of occlusal discrepancies on gingival width. J. Periodontol..

[4] Kao R.T., Pasquinelli K. (2002). Thick vs. thin gingival tissue: A key determinant in tissue response to disease and restorative treatment. J. Calif. Dent. Assoc..

[5] Borghetti A., Monnet-Corti V. Chirurgie Plastique Parodontale. https://www.leslibraires.fr/livre/832321-chirurgie-plastique-parodontale-alain-borghetti-virginie-monnet-corti-cahiers-de-protheses-editions.

[6] Dawson P.E. (2007). Functional Occlusion: From TMJ to Smile Design.

[7] Watanabe-Kanno G.A., Abrão J. (2012). Study of the number of occlusal contacts in maximum intercuspation before orthodontic treatment in subjects with Angle Class I and Class II Division 1 malocclusion. Dent. Press J. Orthod..

[8] Driscoll C.F., Freilich M.A., Guckes A.D., Knoernschild K.L., Mcgarry T.J. (2017). The Glossary of Prosthodontic Terms: Ninth Edition. J. Prosthet. Dent..

[9] American Academy of Periodontology (2001). Glossary of Periodontal Terms. https://www.perio.org.

[10] Fan J., Caton J.G. (2018). Occlusal trauma and excessive occlusal forces: Narrative review, case definitions, and diagnostic considerations. J. Periodontol..

[11] Zucchelli G. (2013). Mucogingival Esthetic Surgery.

[12] Kim D.M., Bassir S.H., Nguyen T.T. (2020). Effect of gingival phenotype on the maintenance of periodontal health: An American Academy of Periodontology best evidence review. J. Periodontol..

[13] Kim D.M., Neiva R. (2015). Periodontal soft tissue non-root coverage procedures: A systematic review from the AAP Regeneration Workshop. J. Periodontol..

[14] Zweers J., Thomas R.Z., Slot D.E., Weisgold A.S., Van der Weijden F.G.A. (2014). Characteristics of periodontal biotype, its dimensions, associations and prevalence: A systematic review. J. Clin. Periodontol..

[15] Cairo F., Nieri M., Cincinelli S., Mervelt J., Pagliaro U. (2011). The interproximal clinical attachment level to classify gingival recessions and predict root coverage outcomes: An explorative and reliability study. J. Clin. Periodontol..

[16] Glickman I., Smulow J. (1965). Further Observations on the Effects of Trauma from Occlusion in Humans. Periodontics.

[17] Glickman I. (1965). Clinical Significance of Trauma From Occlusion. J. Am. Dent. Assoc..

[18] Krishna Prasad D., Sridhar Shetty N., Solomon E.G.R. (2013). The influence of occlusal trauma on gingival recession and gingival clefts. J. Indian Prosthodont. Soc..

[19] Kundapur P.P., Bhat K.M., Bhat G.S. (2009). Association of trauma from occlusion with localized gingival recession in mandibular anterior teeth. Dent. Res. J..

[20] Qadeer S., Kerstein R., Kim R.J.Y., Huh J.B., Shin S.W. (2012). Relationship between articulation paper mark size and percentage of force measured with computerized occlusal analysis. J. Adv. Prosthodont..

[21] Carey J.P., Craig M., Kerstein R.B., Radke J. (2007). Determining a relationship between applied occlusal load and articulating paper mark area. Open Dent. J..

[22] Saad M.N., Weiner G., Ehrenberg D., Weiner S. (2008). Effects of load and indicator type upon occlusal contact markings. J. Biomed. Mater. Res. B Appl. Biomater..

[23] Harper K.A., Setchell D.A. (2002). The use of shimstock to assess occlusal contacs; a laboratory study. Int. J. Prosthodont..

[24] Okeson J. (2003). Management of Temporomandibular Disorders and Occlusion.

[25] Kleinberg I. (1991). Occlusion Practice and Assessment.

[26] Smukler H. (1991). Equilibration in the Natural and Restored Dentition.

[27] Majithia I.P., Arora V., Anil Kumar S., Saxena V., Mittal M. (2015). Comparison of articulating paper markings and T Scan III recordings to evaluate occlusal force in normal and rehabilitated maxillofacial trauma patients. Med. J. Armed. Forces India.

[28] Brizuela-Velasco A., Álvarez-Arenal Á., Ellakuria-Echevarria J., del Río-Highsmith J., Santamaría-Arrieta G., Martín-Blanco N. (2015). Influence of Articulating Paper Thickness on Occlusal Contacts Registration: A Preliminary Report. Int. J. Prosthodont..

[29] Jati A.S., Furquim L.Z., Consolaro A. (2016). Gingival recession: Its causes and types, and the importance of orthodontic treatment. Dent. Press J. Orthod..

[30] Joss-Vassalli I., Grebenstein C., Topouzelis N., Sculean A., Katsaros C. (2010). Orthodontic therapy and gingival recession: A systematic review. Orthod. Craniofac. Res..

[31] Bin Bahar B.S.K., Alkhalidy S.R., Kaklamanos E.G., Athanasiou A.E. (2020). Do orthodontic patients develop more gingival recession in anterior teeth compared to untreated individuals? A systematic review of controlled studies. Int. Orthod..

[32] Kerstein R.B., Radke J. (2014). Clinician accuracy when subjectively interpreting articulating paper markings. Cranio.

[33] Lo Giudice R., Famà F. (2020). Health Care and Health Service Digital Revolution. Int. J. Environ. Res. Public Health.

